# Top-Gated P-MOSFET with CVD-Grown WSe_2_ Channels via Self-Aligned WO_*x*_ Conversion for Spacer Doping

**DOI:** 10.1021/acs.nanolett.5c00813

**Published:** 2025-04-15

**Authors:** Meng-Zhan Li, Terry Y. T. Hung, Wei-Sheng Yun, D. Mahaveer Sathaiya, Sui-An Chou, San Lin Liew, Ying-Mei Yang, Kuang-I Lin, Tung-Ying Lee, Chao-Ching Cheng, Chung-Cheng Wu, Iuliana P. Radu, Minn-Tsong Lin

**Affiliations:** † Department of Physics, National Taiwan University, Taipei 10617, Taiwan; ‡ Taiwan Semiconductor Manufacturing Company, Hsinchu 308001, Taiwan; § National Cheng Kung University, Tainan 70101, Taiwan; ∥ Institute of Atomic and Molecular Sciences, Academia Sinica, Taipei 10617, Taiwan; ⊥ Research Center for Applied Sciences, Academia Sinica, Taipei 11529, Taiwan

**Keywords:** low-dimensional materials, WSe_2_, converted tungsten oxide, p-doping, MOSFET, self-aligned

## Abstract

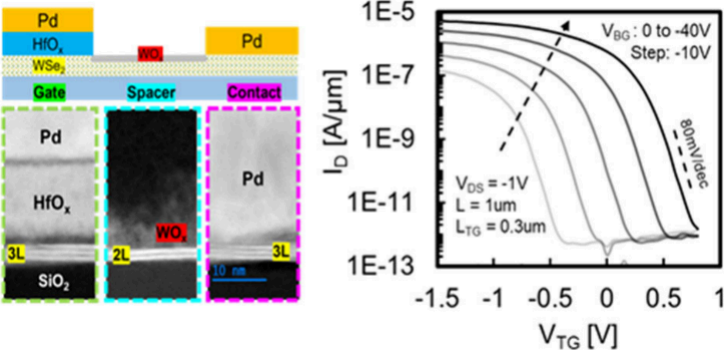

WO_*x*_ conversion for doping is highlighted in recent advancements in WSe_2_ p-FETs. While past studies focused on exfoliated WSe_2_ flakes, our research examines CVD-grown WSe_2_ films, assessing the impact of this doping on channel mobility and contact resistance in devices. Our approach enables effective threshold voltage tuning in both n- and p-type FETs with various low-dimensional material channels with the doping mechanism well captured by TCAD simulations. When applied to WSe_2_, trilayer devices exhibited a good and comparable median field-effect mobility of 65 cm^2^/V·s following the conversion process. Consequently, trilayer WSe_2_ was used to demonstrate top-gated p-MOSFETs via self-aligned WO_*x*_ conversion in the spacer region, achieving a 250-fold enhancement in the on-current while maintaining a subthreshold swing of 80 mV/dec. Our findings provide a comprehensive understanding of WO_*x*_ conversion and its general applicability, paving the way for its use in future logic devices with low-dimensional materials.

Semiconducting low-dimensional (LD) materials, such as one-dimensional carbon nanotubes (1D-CNTs) and two-dimensional transition-metal dichalcogenides (2D-TMDs), have potential as channel material candidates due to their ultrathin and dangling bond-free nature, allowing aggressive device scaling without compromising the mobility.^1^ However, Fermi-level pinning is a critical bottleneck in LD materials,^2^ resulting in high contact resistance (*R*_C_) and undesired ambipolar transport.^3,4^ As a result, realizing CMOS with these materials is hindered where unipolar transport and depletion mode are required for minimizing the power consumption. To address these issues, doping approaches, along with suppressing ambipolar transport, are crucial. To date, a stable, damage-free, and fab-compatible doping approach for LD materials, particularly p-doping, is still limited. Surface modification by molecules with strong oxidizing tendency, such as nitric oxide (NO_*x*_)^5,6^ or gold chloride (AuCl_*x*_),^7,8^ can be very effective p-doping methods. However, their stability and processing capability remain to be examined. In addition, molecule absorption on the surface may lead to the creation of scattering sites, which could potentially degrade channel mobility. Such an issue may also be encountered in substitutional doping approaches.^9,10,40^ On the other hand, remote doping approaches by depositing high work function oxides^11−14^ enable p-doping effects without degrading its mobility. Recently, converted tungsten oxide (converted-WO_*x*_) from the top surface of WSe_2_ is widely noted.^15−26^ By exposing WSe_2_ to gentle O_2_ plasma or UV ozone, the topmost layer of WSe_2_ is converted to a thin WO_*x*_ layer. The high work function^23,38^ of such a layer enables p-doping to the underlying channel through band realignment. While most literature works demonstrate this WO_*x*_ conversion on exfoliated WSe_2_ flakes or semimetallic graphene,^23,24^ applying it to CVD-grown films, including various kinds of LD materials with the potential for future applications, is still limited. Furthermore, basic features, such as self-limitation, conversion thickness, and the electrical impacts on the underlying channel materials, require thorough examination. For example, the device metrics after implementing WO_*x*_ conversion on WSe_2_ with extremely scaled thicknesses (≤3 layers) remain largely unexplored. This is of significant interest because this thickness regime coincides with substantial band structure evolution.^28,29^ Notably, as layer number increases from 1L to 3L, the bandgap diminishes, approaching bulk-TMD values. This bandgap reduction, along with changes in band edges and transport valleys,^30^ is expected to influence critical electrical parameters—*V*_TH_ shifting, carrier mobility, Schottky barrier height (SBH), and *R*_C_—in 2D WSe_2_ devices with ultrathin channels. The consideration of layer number and bandgap has recently been shown to be important in substitutional doping,^40^ as the doping efficacy is layer-thickness-dependent. Furthermore, future device development always necessitates ultrathin channels for superior gate control. Therefore, studying doping in the ultrathin layer regime is essential for optimizing the 2D device performance.

Here, we extensively investigate the viability of utilizing converted-WO_*x*_ as a p-doping approach for technologically relevant LD channel materials, exhibiting varying bandgaps due to different material types or varying layer numbers. Our approach, a self-limiting process, allows tuning of the *V*_TH_ positions by forming an atomically thin WO_*x*_ layer on top of various LD field-effect transistors (FETs), including p-type 1L-WSe_2_, n-type 1L-MoS_2_, and p-type carbon nanotube (CNT). TCAD modeling further supports each doping strength, according to the individual band alignment and corresponding acceptor level and concentration. Subsequently, we benchmarked the transport properties of WSe_2_ channels with different layer numbers, determining that 3L-WSe_2_ is optimal for converted-WO_*x*_ p-doping. Furthermore, we experimentally observe an *R*_C_ increase for 2L-WSe_2_ after the conversion process, which contrasts with the *R*_C_ improvement reported in most of the literature. Finally, we demonstrate the enhanced performance of a 3L-WSe_2_ p-MOSFET, achieved by forming WO_*x*_ at spacer regions in a self-aligned manner. Our findings elucidate the use of WO_*x*_ conversion as a versatile p-doping strategy for LD materials, facilitating their advancement in logic electronic applications.

In the presented study, the transfer process of CVD-grown 1L-WSe_2_ onto a target LD material is a crucial step in the exploration of converting WSe_2_ to WO_*x*_ for various applications. Here, WSe_2_ is chosen as the starting material for the conversion process, since it could exhibit the best self-limiting ability to protect the underlying channels.^18,24,26^ The transferring quality of 1L-WSe_2_ can directly determine the flatness/uniformity of the converted-WO_*x*_ layer. Here, we employ a wet-transfer process flow: PMMA/1L-WSe_2_ stack is first partially detached from the growth sapphire substrate with an ammonia etchant and then released in deionized water (DIW) to remove the remaining etchant. Figure 1a depicts the following wet-transfer process where the PMMA/1L-WSe_2_ stack is scooped up by the target LD material on a substrate. Normally, wet-transfer process is usually accomplished in a DIW environment. Once hydrophobic materials, such as wafer-scale LD materials on an oxide substrate, are used to scoop up the PMMA/1L-WSe_2_ floating on DIW, the presence of high surface tension can not only cause cracks and wrinkles to the monolayer^31^ but also peel off the target LD materials. To overcome this issue, we added isopropanol (IPA) into to DIW, which effectively reduces the originally high surface tension.^32^ A trade-off was found between the formation of wrinkles and sinking of PMMA due to the insufficient buoyancy, as adjusting the mixing ratio of IPA/DIW (Figure S1). The optimized volume ratio of IPA:DIW was determined to be 1:5, which corresponds to 13.6 wt %. With this mixture of IPA/DIW, the surface tension and contact angle is reduced while preventing the PMMA sinking, as shown in Figure S2. This resulted in smoother stacking materials and good conformity of converted-WO_*x*_, as seen in the representative TEM image in Figure 1b.

**Figure 1:**
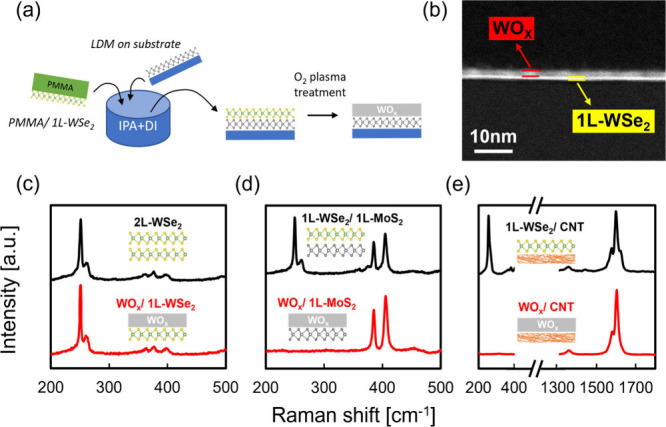
**Universal WO**_*x*_**p-doping approach on LD materials.** (a) Schematic of the process flow. (b) TEM image showing uniform and atomically thin WO_*x*_ on top of a monolayer (1L) of WSe_2_. Raman spectra of 1L-WSe_2_ transferred onto (c) 1L-WSe_2_, (d) 1L-MoS_2_, (e) CNT (black) and after WO_*x*_ conversion by O_2_ plasma (red).

Prior to investigating the electrical properties, nondestructive optical characterizations were employed to confirm the conversion of the topmost layer of WSe_2_ without damaging the underlying materials. Figure 1c shows that the primary peaks, E_2g_ and A_1g_, of WSe_2_ display similar intensity and full width at half-maximum (fwhm) both before and after conversion. Figure 1d shows a similar result for 1L-MoS_2_, where only the characteristic peaks of 1L-WSe_2_ vanished after conversion. Moreover, the conversion process was also tested on carbon nanotubes (CNTs), which are highly susceptible in the oxygen plasma environment, as shown in Figure 1e. Results demonstrated no change in either the G or 2D peaks, and the widely used method for detecting defect formation (D/G ratio of CNTs)^33^ also showed no apparent increase, as shown in the Figure S3b. Consequently, a self-limiting process was established and proven to be general for various LD materials. Furthermore, Figure S3d shows an AFM image of a CNT device partially covered by WO_*x*_ which was converted from 1L-WSe_2_. In the region covered by converted-WO_*x*_ and S/D contacts, the morphology of the CNT network was observable, whereas the exposed region showed a plain surface, indicative of an etching effect. This result also revealed the potential for WO_*x*_ conversion to be implemented as a self-aligned process.

To showcase how converted-WO_*x*_ can change the underlying LD materials’ electronic behaviors, 1L-WSe_2_ was transferred onto the investigated back-gate FET devices with three kinds of LD channels, including unipolar n-type CVD-grown 1L-MoS_2_, ambipolar CVD-grown 1L-WSe_2_, and p-favored CNT. Here, we defined the position of *V*_TH_ at *I*_D_ = 100 nA based on the constant-current method. We noted that the selection of such a current level did not change the result much, considering parallel shifting of the transfer curves (see Figure S4). For CVD-grown 1L-MoS_2_ n-FET, normally on behavior is typically observed due to unintentional doping during the growth or the fabrication process.^34^ However, in Figure 2a, we show that the normally on behavior is turned into normally off by having a positive *V*_TH_ shifting (Δ*V*_TH_) ∼ 37 V induced by the p-doping effect of converted-WO_*x*_. This shifting can be utilized not only to solve the high standby power consumption in CMOS, but also to provide multi-*V*_TH_ choices for different applications. Similarly, as shown in Figure 2b of 1L-WSe_2_ p-FET, positive Δ*V*_TH_ ∼ 32 V is observed, and its polarity is changed from ambipolar to CMOS favored unipolar behavior. Moreover, we show that this p-doping effect is even stronger on 1D-CNT. In Figure 2c, a significant positive Δ*V*_TH_ of ∼ 59 V is observed. We also observe the worsen subthreshold swing (S.S.) after conversion, which may be attributed to the high doping density close to degenerate level. To offer a clearer insight for converted-WO_*x*_ p-doping on different LD materials, we perform TCAD modeling, considering: (i) 2DMs and CNT band parameters with 2D and 1D-DoS charge model respectively, (ii) energy dependent interface trap charge model and (iii) Fermi level at the midgap as the feature of off-state, for extracting the charge-transfer doping (CTD) from the experimental Δ*V*_TH_. This model extracts CTD, from Δ*V*_TH_ of converted-WO_*x*_ on n-type 1L-MoS_2_, p-type 1L-WSe_2_ and p-type CNT, in the range of 0.7 to 1.4 × 10^13^ cm^–2^ as shown in Figure 3d–f. Also shown is the extracted acceptor defect energy distribution at the converted-WO_*x*_ LD semiconductors interface. Parameters for TCAD modeling are summarized in Table S1.

**Figure 2:**
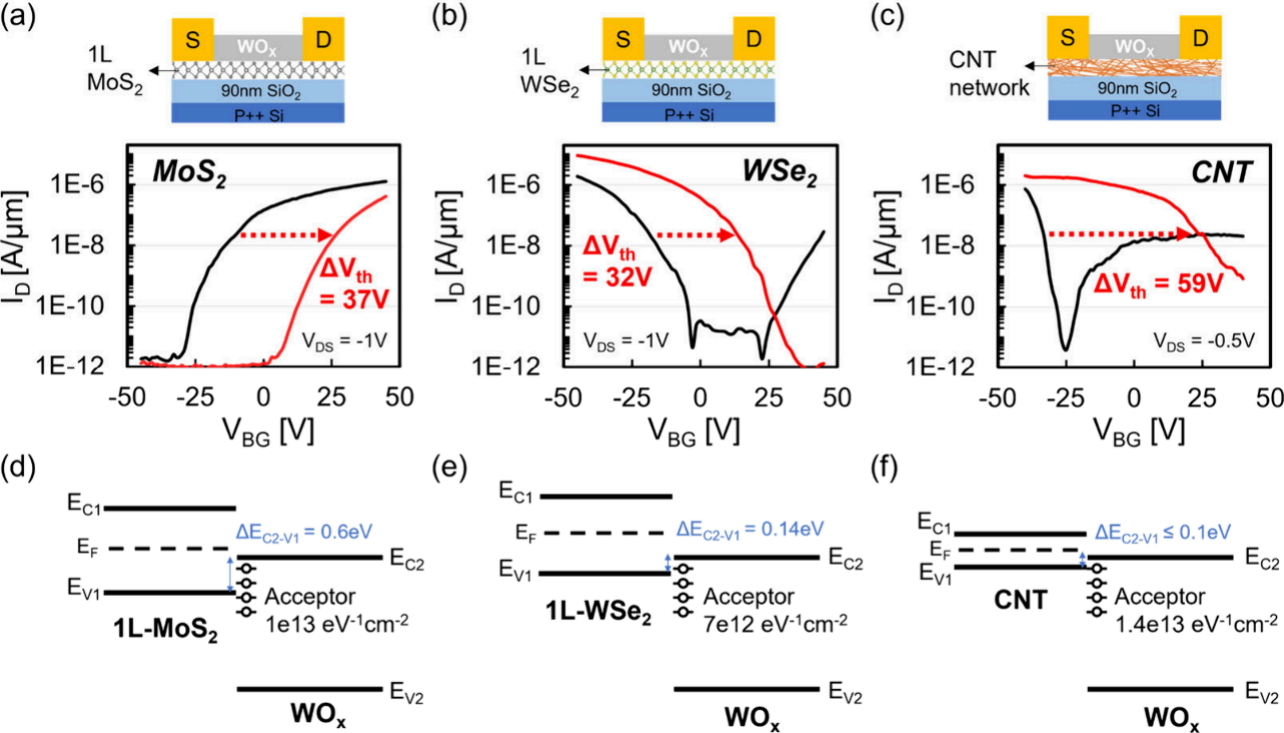
**Performing converted-WO**_*x*_**p-doping on different kinds of low-dimensional devices.** Transfer curves of (a) 1L-MoS_2_, (b) 1L-WSe_2_, (c) CNT network of before (black)/after (red) WO_*x*_ conversion. (d–f) Corresponding band alignment schematics. All devices used Pd contacts and had the same channel length of 2 μm.

**Figure 3:**
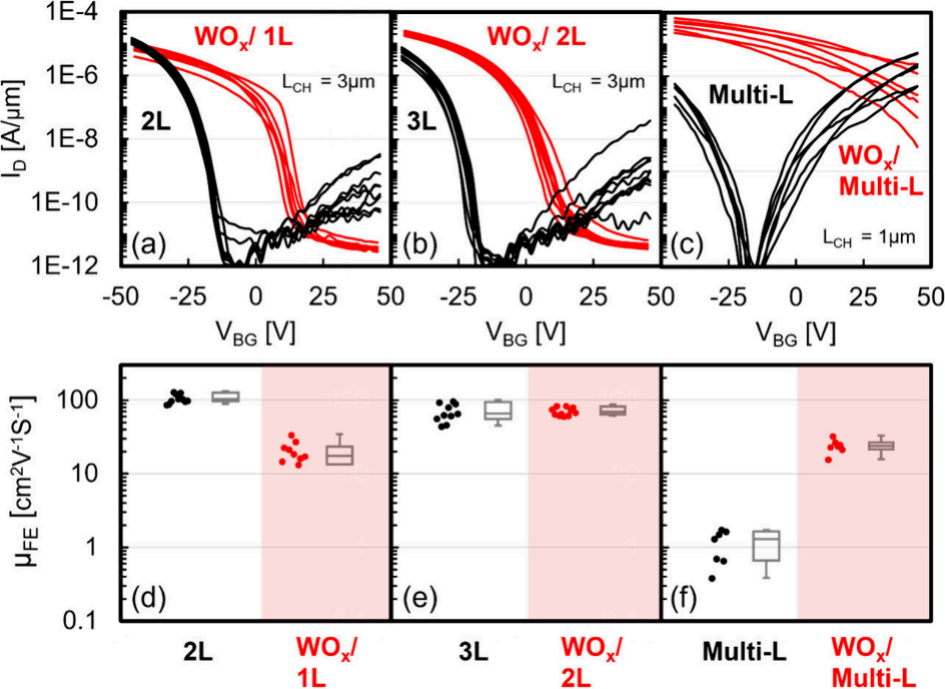
**Converted-WO**_*x*_**p-doping layer on various layer number of WSe**_**2**_**.** Transfer curves of two-probe devices based on (a) 2L-, (b) 3L-, and (c) multi- (Multi-L) WSe_2_ before (black)/after (red) WO_*x*_ conversion. Note that conversion will consume the topmost 1L-WSe_2_, resulting in a 1L reduction of the channel. *V*_DS_ = −1 V was used for all of the devices. (d–f) Corresponding statistics of the calculated field-effect mobility in (a–c).

WSe_2_ has special potential in p-FET application due to its relatively higher valence band position among semiconducting TMDs, in favor of realizing the smaller barrier height for p-type contact. In practical device applications, different layer numbers of WSe_2_, as long as they are obtainable by the CVD approach, can potentially be utilized as the p-type channel material. TMDs often associate bandgap and band edge positions with the number of layers and the strength of interlayer coupling, and the narrower band gap can be achieved in few-layer or bulk TMDs in the presence of interlayer coupling. Accordingly, for WSe_2_ with a layer number more than one, the hole Schottky barrier height could be reduced by the higher valence band edge compared to 1L-WSe_2_. However, thicker channel thickness usually sacrifices the gate controllability as the device is scaled,^35^ despite the benefits owing to the smaller bandgap. Here, we implement WO_*x*_ conversion on 2L-/3L- and multi- (multilayer) WSe_2_, aiming to reveal the core differences of this doping approach in terms of bandgap dependency. Note that the conversion process consumes the topmost layer of WSe_2_, so the 2L/3L samples are finished with 1L/2L, alongside the onset to trigger interlayer coupling. Since achieving a uniform and wafer-scale CVD-grown 2L/3L-WSe_2_ film has not yet been accomplished to the best of our knowledge, instead, we repeat monolayer transferring, which creates a deterministic number of layers with high coverage films. To strengthen the interlayer coupling in the as-stacked 2L- and 3L-WSe_2_ films, 400 °C annealing was performed in a high vacuum at 10^–7^ Torr for 2 h. Detailed discussion and characterization of the stacking process can be found in Figure S5. Here, multi-WSe_2_ was prepared by mechanical exfoliation, providing a connection between our findings based on CVD-grown thin films and the results reported in previous literature reports based on exfoliated flakes.

In Figure 3a–c, we showcase the transfer characteristics of 2L-, 3L- and multi-WSe_2_ two-probe devices before and after performing WO_*x*_ conversion, i.e., converted-WO_*x*_/1L-WSe_2_, converted-WO_*x*_/2L-WSe_2_ and converted-WO_*x*_/multi-WSe_2_. Apparently, multi-WSe_2_ can obtain the highest doping level, even close to being degenerate. This is because multi-WSe_2_ has the smaller bandgap compared to the thinner WSe_2_.^17,28^ For 2L- and 3L-WSe_2_, the originally ambipolar transport properties become unipolar by eliminating the n-branch after the conversion process. Comparing the doping level between both cases, a slightly higher medium value for 2L-WSe_2_ (Figure S6) could indicate a smaller bandgap nature. The trend of the doping level also implies that the doping effect starts to be dominant as the bandgap narrows, where the valence band maximum of WSe_2_ moves closer to the conduction band minimum of WO_*x*_.

When designing electronic devices, on-state current (*I*_ON_) and *V*_TH_ are two important factors to be considered. To adjust *V*_TH_ to a suitable position, doping is employed. However, doping could be beneficial or adverse to *I*_ON_. If channel mobility is fixed, doping, which increases carrier density, should reduce channel resistance and thus increase *I*_ON_. Nevertheless, doping level is often associated with the mobility change. For instance, high doping level may enhance charged impurity scattering and short-range scattering which lead to mobility degradation.^8,27^ Consequently, evaluation of the changes in channel mobility before and after doping is essential to ensure that the *I*_ON_ performance remains consistent. In Figure 3d–e, we show the statistics of field-effect mobility (*μ*_FE_) extracted from the transfer curves in Figure 3a–b, using the equation , where *L*/*W* is the length/width of the two-probe devices and *C*_ox_ is the back-gate capacitance. Here, *C*_ox_ is calculated to be 38.4 nF/cm^2^ from the back-gate dielectric of 90 nm SiO_2_. Different trends were observed in *μ*_FE_ change after the conversion process: increased in multi-WSe_2_ (converted-WO_*x*_/multi-WSe_2_), equal in 3L-WSe_2_ (converted-WO_*x*_/2L-WSe_2_) and decreased in 2L-WSe_2_ (converted-WO_*x*_/1L-WSe_2_). For multi-WSe_2_, the increase of *μ*_FE_ is likely attributed to the lowering of *R*_C_. As a previous literature work suggested,^16^ WO_*x*_ can “sneak-in” between the contact metal and WSe_2_, modifying the contact property. Since the bandgap of multi-WSe_2_ is almost fixed after converting the topmost 1L, the “sneak-in” WO_*x*_ can reduce the SBH by increasing the effective work function of contact. On the other hand, performing WO_*x*_ conversion on 2L-WSe_2_ can lead to a drastic bandgap change from a an originally indirect/small one to a direct/large one, because of the elimination of interlayer coupling. In this case, both the band alignment between metal/WSe_2_ and the effective mass of carriers in WSe_2_ could change, which could respectively alter the *R*_C_ and channel mobility. Interestingly, no *μ*_FE_ change was revealed between 3L-WSe_2_ and the subsequent converted-WO_*x*_/2L-WSe_2_, maintaining a high median of ∼65 cm^2^/V·s. This observation possibly indicates a threshold layer number for proper implementation of converted-WO_*x*_ p-doping on WSe_2_.

In most cases, field-effect mobility (*μ*_FE_) is regarded as the representative of channel mobility and used to quantify the overall channel behavior after specific treatments such as doping,^8,27^ capping,^36,37^ etc. However, calculation of *μ*_FE_ usually includes the impact of *R*_C_ which is typically nonnegligible for Schottky-type 2D TMDs FET, causing the unmatching between *μ*_FE_ and intrinsic channel mobility (*μ*_int_). Sometimes, contacts may exhibit unequal or even opposing changes compared with the channel after a specific treatment, leading to an erroneous conclusion solely considering *μ*_FE_. Therefore, decoupling the *R*_C_ effect from the mobility estimation is the primary criterion for assessing channel changes. In our case, degradation of *μ*_FE_ observed for 2L-WSe_2_ being converted into WO_*x*_/1L-WSe_2_ could be attributed two possibilities: (i) *R*_C_ is increased, and (ii) *μ*_int_ is reduced after conversion. Here, we employed four-probe measurement^39^ to separate the contribution of channel/ contact in a 2L-WSe_2_ device before/after the conversion process. Figure 4a shows our device design for the four-probe measurement, in which pairs of probes at both sides allow the sensing of a voltage drop in the channel. In Figure 4b, transfer curves before/after the conversion process are shown. A clear *V*_TH_ shifting indicates a p-doping effect by the formation of WO_*x*_, while the maximum achievable *I*_ON_ is slightly lower in the converted sample. Figure 4c shows the field-effect mobility extracted from the transfer curves and the intrinsic mobility by four-probe measurement, versus the carrier concentration (*N*_h_) determined by the *V*_TH_ of the channel (see Figure S7 for the extraction). After the conversion process, different degrees of reduction in *μ*_FE_ and *μ*_int_ were observed. While the *μ*_FE_ reduced up to 37% comparing the peak positions, *μ*_int_ is only 13% off. Thus, we conclude that the excess reduction for *μ*_FE_ should be due to the impact of *R*_C_. Indeed, in Figure 4d, where we plot *R*_C_ versus *N*_h_, a 30% increase of *R*_C_ was revealed after the conversion process at the peak positions to extract *μ*_FE_. As previously discussed, a predominant bandgap difference happens as layer number changes from 2L to 1L for TMDs. Figure 4e schematically illustrates the band alignment at the contact before and after the conversion process. Accordingly, the increase of *R*_C_ is likely due to the enlarged SBH by the deeper valence band edge as the channel layer shrinks down to 1L, stemming from an inherent property of 2D-TMDs. Therefore, we performed a temperature-dependent *I*_D_–*V*_BG_ measurement to experimentally extract the SBH of a 2L-WSe_2_ device before and after WO_*x*_ conversion. As shown in Figure S8, the extracted SBH increased from 25 to 62 meV after conversion. This increase confirms our hypothesis that the increased *R*_C_ is due to the enlarged SBH caused by the band edge shifting. Lastly, we comment that our best achieved *R*_C_ of ∼20 kΩ·μm in the pristine 2L-WSe_2_ still has room for improvement. To reduce the specific contact resistivity, ensuring a uniform metal-2D interface and a large grain size of the contact metal could be a feasible strategy.^26^

**Figure 4:**
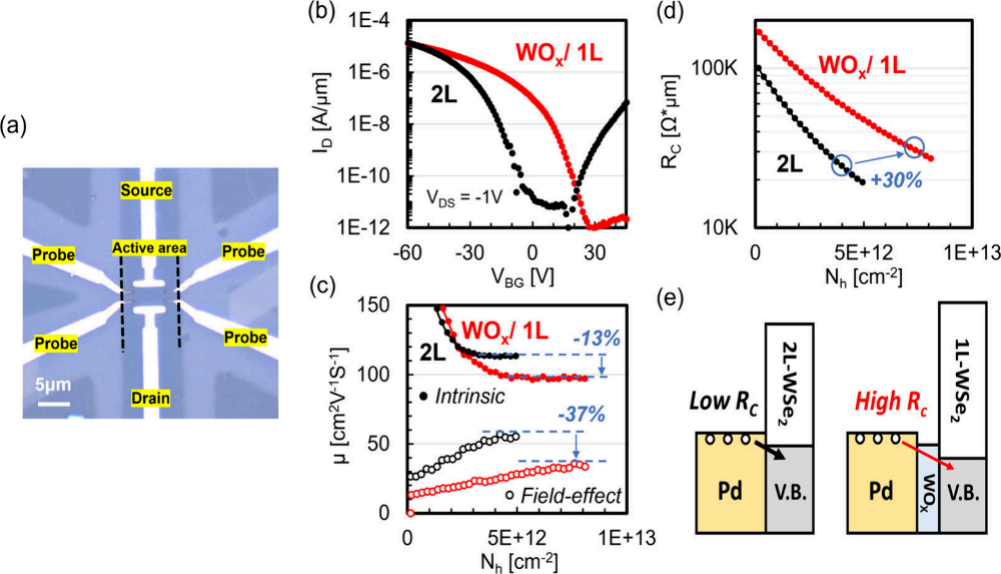
**Four-probe measurement on a 2L-WSe**_**2**_**device before and after forming converted-WO**_*x*_**p-doping layer.** (a) OM image of a four-probe device. (b) Transfer curves before (black) and after (red) conversion, (c) intrinsic (solid symbol) and field effect (empty symbol) mobility versus carrier, (d) *R*_C_ versus carrier, and (e) schematics of band alignment at the contact before and after WO_*x*_ conversion, where 2L-WSe_2_ is converted to WO_*x*_/1L-WSe_2_.

In device operation, *R*_C_ and *μ*_int_ effects are entangled, and only the combination of both, *μ*_FE_, is an explicit property. We have demonstrated that performing the WO_*x*_ conversion process on WSe_2_ with a layer number ≥3L can prevent the *μ*_FE_ degradation issue. However, by means of gate controllability, the channel thickness needs to be as thin as possible. In Figure 3e, 3L-WSe_2_ shows a good and comparable median *μ*_FE_ ∼ 65 cm^2^/V·s following the conversion process. Thus, 3L could be a balanced layer number for demonstrating an efficient doping effect by the converted-WO_*x*_ p-doping approach without sacrificing *μ*_FE_, or *R*_C_, while maintaining good gate-controllability. The MOSFET structure with spacers on both sides of an underlapped top gate enables self-aligned spacer doping by the WO_*x*_ conversion technique, as shown in Figure 5a. Fabrication of an underlapped MOSFET structure started from fabricating a two-probe device with Pd S/D contacts, patterned by e-beam lithography. Then, e-beam lithography was utilized again to pattern the top-gate electrode, followed by low-temperature ALD of HfO_*x*_ and e-beam evaporation of Pd as the gate metal. The SEM image of the final device is provided in Figure S9. The conversion process was then performed to convert 3L-WSe_2_ into WO_*x*_/2L-WSe_2_ in the spacer region. In Figure 5b, TEM images of the top-gate, spacer, and contact region after WO_*x*_ conversion are shown, where the HfO_*x*_ gate dielectric is revealed to be ∼9 nm and a 1L reduction at the spacer region is observed. Figure 5c,e shows the transfer characteristics of top-gate sweeping before/after WO_*x*_ conversion at a given back gate voltage. Reducing contact/spacer resistance and improving subthreshold swing (S.S.) are achieved by narrowing the tunneling distance in Schottky barrier and eliminating the superposition of different *V*_TH_ between contact and channel, respectively. *I*_ON_ is enhanced more than 2 orders of magnitude at the same combination of V_TG_ and *V*_BG_, and an approximately 250-fold increase is observed in the maximum achieved *I*_ON_. Sub-100 mV/dec of S.S. is sustained over more than two decades of current. Note that spacer doping fixes the Fermi level in both contact and spacer regions, eliminating the superposition of different *V*_TH_ and enabling a wider operating range with a minimum S.S. of ∼80 mV/dec. EOT of the top gate is extracted to be 2.6 nm based on a similar slope at the S.S. region with different *V*_BG_ after spacer doping. Utilizing the unique properties of WO_*x*_ p-doping through conversion and underlapped top gate structure, a MOSFET enabled by self-aligned spacer p-doping is demonstrated, addressing the challenges in *R*_C_ and S.S. for 2D-TMD p-FETs.

**Figure 5:**
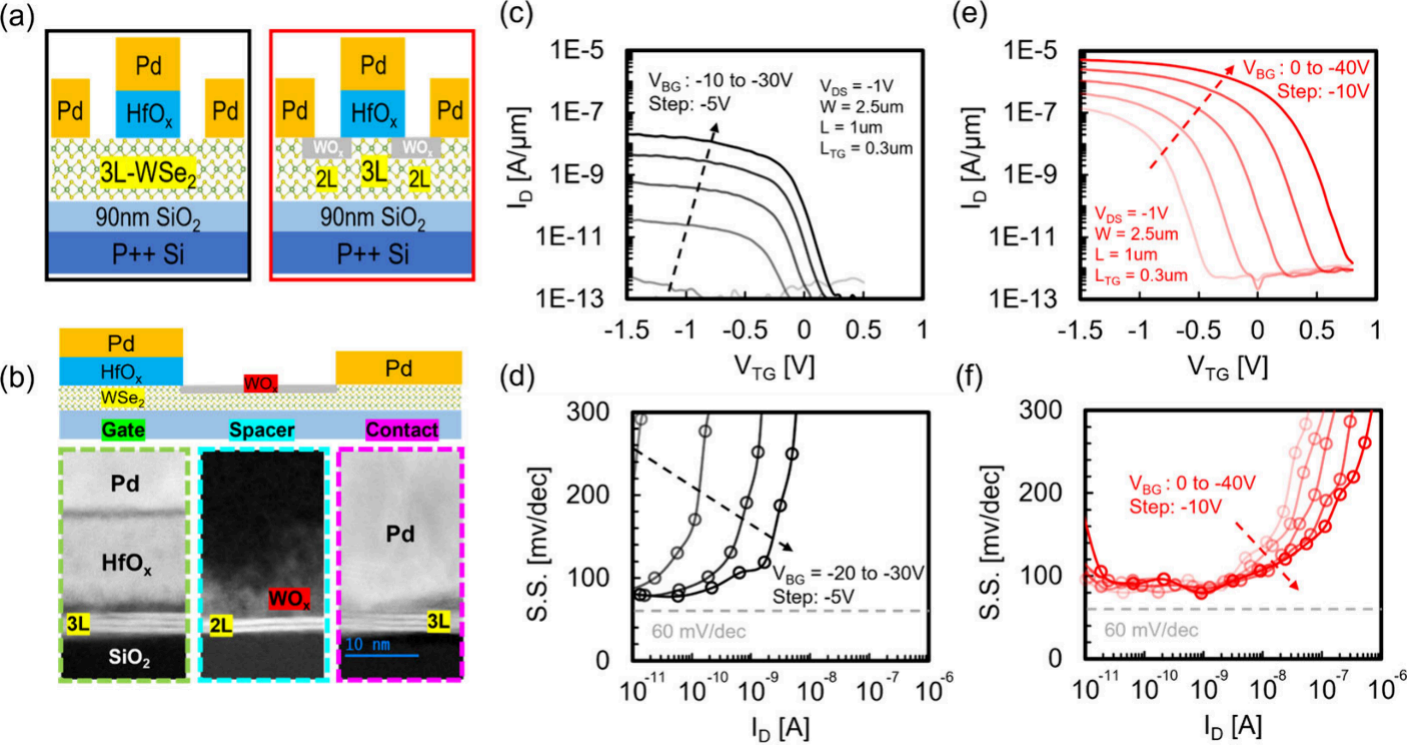
**3L-WSe**_**2**_**p-MOSFET device utilizing WO_*x*_ conversion as self-aligned spacer doping.** (a) Schematics of device structure before (left) and after (right) WO_*x*_ conversion process. (b) TEM images of the top-gate, spacer and contact region after WO_*x*_ conversion. Top-gate transfer characteristics (c) before and (e) after WO_*x*_ conversion were determined under various *V*_BG_. S.S. versus *I*_D_ of (d) before and (f) after WO_*x*_ conversion under various *V*_BG_.

In conclusion, a comprehensive study of converted-WO_*x*_ by 1L-WSe_2_ has been conducted to highlight the universal p-doping ability in devices and to delve into the underlying doping mechanism. We successfully implemented this doping technique on LD n-/p-FETs, including 2D-MoS_2_, 2D-WSe_2_, and 1D-CNT, by transferring CVD-grown monolayer WSe_2_ followed by O_2_ plasma conversion. In addition, the study of layer number dependence revealed that 3L-WSe_2_, after WO_*x*_ conversion, exhibits a noticeable positive *V*_TH_ shifting while maintaining a high μ_FE_ ∼ 65 cm^2^/V·s and a large on–off current ratio in devices. Subsequently, WO_*x*_ conversion was applied to a top-gated 3L-WSe_2_ p-MOSFET to achieve a self-aligned p-doping process in the spacer, demonstrating enhanced *I*_ON_ performance with a decent S.S. of ∼80 mV/dec over a few decades. This result indicates the feasibility of the converted-WO_*x*_ p-doping approach for future logic electronic applications utilizing LD materials.

## Supporting Information Available

Adding IPA into DIW to reduce the surface tension; demonstrations of transferring using IPA/DIW liquid; performing WO_*x*_ conversion on CNT network; parameters used in the TCAD modeling of CTD mechanism; parallelly shifted transfer characteristics of doped 1L-MoS_2_/WSe_2_; detail stacking process; PL spectra of 1L-WSe_2_ and stacked 2L-WSe_2_; *V*_TH_ shifting for 2L-/3L-WSe_2_ FETs after WO_*x*_ conversion; *V*_TH_ determination from four-probe extracted channel conductivity; SBH measurement for 2L-WSe_2_ before/after conversion process; SEM images of the p-MOSFET (PDF)
