# Supplementary material for: Top-Gated P-MOSFET with CVD-Grown WSe2 Channels via Self-Aligned WOx Conversion for Spacer Doping
Source: Nano Lett. 2025 Apr 15;25(17):7037–43. doi: 10.1021/acs.nanolett.5c00813 (PMC12046599; doi:10.1021/acs.nanolett.5c00813)
Supplement: Supplementary file 1 — nl5c00813_si_001.pdf [file nl5c00813_si_001.pdf]

Supporting Information

Top-Gated P-MOSFET with CVD-Grown WSe_2 Channels via Self-Aligned WO_x Conversion for Spacer Doping

Meng-Zhan Li[12], Terry Y.T. Hung[2], Wei-Sheng Yun[2], D Mahaveer Sathaiya[2], Sui-An Chou[2], San Lin Liew[2], Ying-Mei Yang[3], Kuang-I Lin[3], Tung-Ying Lee[2], Chao-Ching Cheng[2], Chung-Cheng Wu[2], Iuliana P. Radu*[2], and Minn-Tsong Lin*[145]

[1]*Department of Physics, National Taiwan University, Taipei 10617, Taiwan*

[2]*Taiwan Semiconductor Manufacturing Company, Hsinchu 308001, Taiwan*

[3]*National Cheng Kung University, Tainan 70101, Taiwan*

[4]*Institute of Atomic and Molecular Sciences, Academia Sinica, Taipei 10617, Taiwan*

[5]*Research Center for Applied Sciences, Academia Sinica, Taipei 11529, Taiwan*

*To whom correspondence should be addressed.

Email: IRADU@tsmc.com (Iuliana P. Radu), mtlin@phys.ntu.edu.tw (Minn-Tsong Lin)

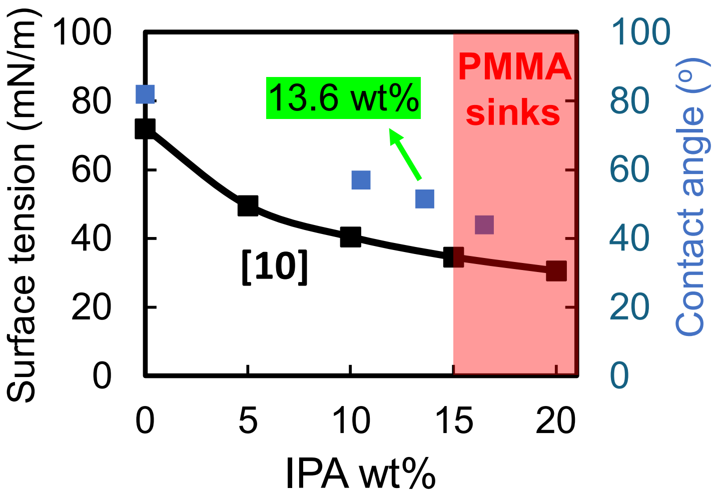

Figure S1. Adding IPA into DIW to reduce the surface tension (adapted from [10]) and the experimentally observed contact angle on 90 nm SiO_2 substrate. In the red-marked region, PMMA cannot float on the liquid level due to the insufficient buoyancy. Near critical value 13.6 wt% is thus to be the optimized ratio for wet-transferring process.

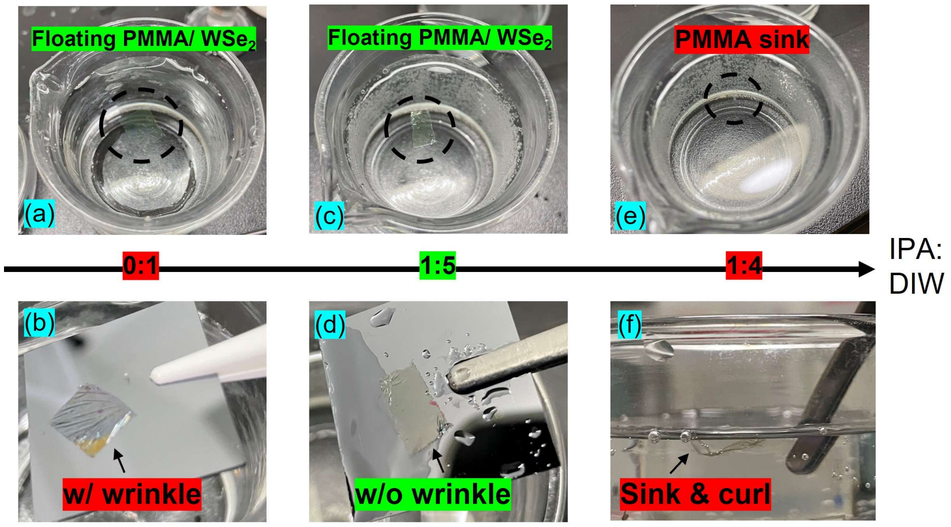

Figure S2. Demonstrations of transferring using IPA/ DIW liquid with different mixing volume ratio. (a)(b) pure DIW, with observable wrinkles. (c)(d) 1:5, smooth film on the substrate, and (e)(f) 1:4, PMMA sink below the liquid level and curled.

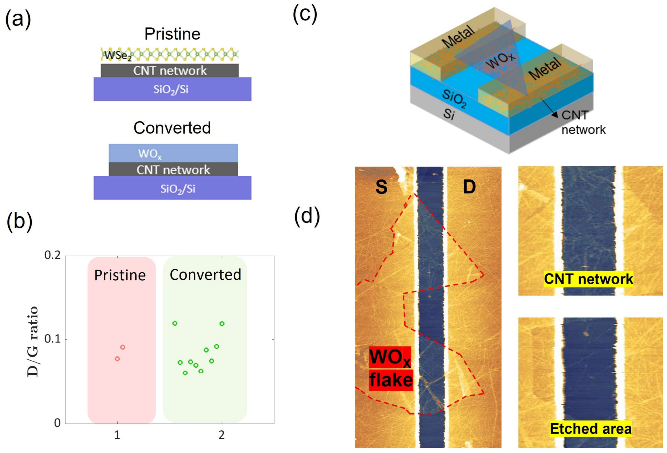

Figure S3. Performing WO_x conversion on CNT network which is highly susceptible to O_2 plasma. (a) Schematic of the sample (1L-WSe_2 on CNT network) before/ after conversion process. (b) Raman D/G ratio used to examine defect formation. No noticeable change is found for the converted case. (c) Device schematic of a converted-WO_x partially capped CNT network device. (d) AFM image shows the CNT network exist under converted-WO_x, while the exposed area is etched during the conversion process.

	Affinity (eV)	Eg (eV)	Dielectric const.
WO_X	5.2eV	2.8	20
1L-MoS_2	4	1.8	6.3
1L-WSe_2	3.7	1.64	6.3
CNT	4.7	0.6	6

Table S1. Parameters used in the TCAD modeling of CTD mechanism.

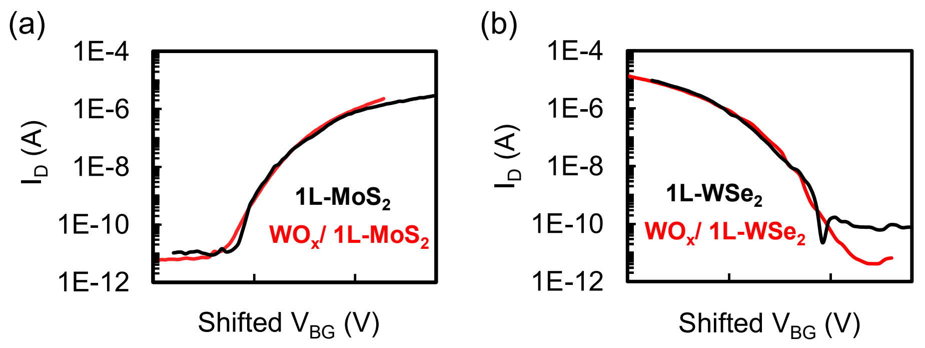

Figure S4. Parallelly-shifted transfer characteristics to match the same V_{TH} position. (a) 1L-MoS_2 (b) 1L-WSe_2 before and after capped with converted-WO_x.

[Detail stacking process]

To strength the interlayer coupling in the as-stacked films, 400 °C annealing was performed in a high vacuum at 10^{-7} torr. Photoluminescence measurement was used to verify the effect of annealing, as shown in Figure S5a-b. It was found that the relative peak intensity of the stacked bilayer, relative to their monolayer counterpart on the same chip, got quenched after annealing, indicating the enhanced interlayer coupling from two weakly coupled monolayers to a properly coupled bilayer. In Figure S5c, tighter distribution of the peak position and normalized peak intensity further suggests good uniformity of interlayer coupling inside the annealed stacked bilayer structure. Additionally, monolayer did not exhibit any apparent change after preformed the same condition of annealing, suggesting the annealing process would not cause the degradation of film quality.

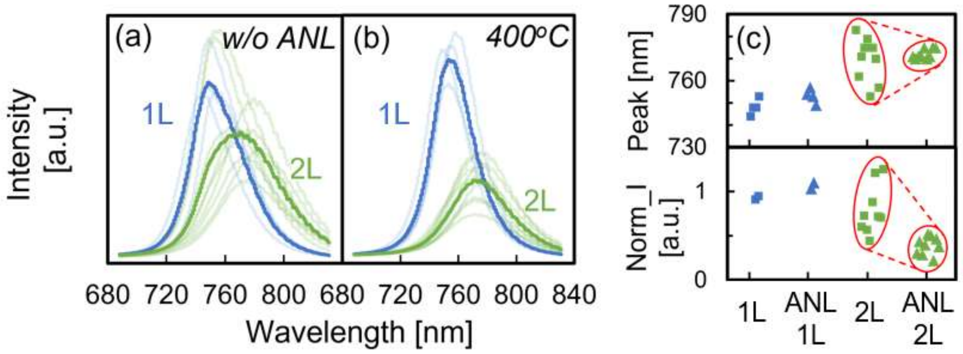

Figure S5. PL spectra of 1L-WSe_2 and stacked 2L-WSe_2 (a) w/o, (b) w/ 400°C vacuum ANL, and the statistics of (c) peak position and intensity normalized to the mean value of 1L counterpart. Reduction in the variation of peak position and the relative intensity for the annealed stacked 2L-WSe_2 suggest the overall enhanced interlayer coupling to produce quenched/ red-shifted PL spectra owing to indirect bandgap.

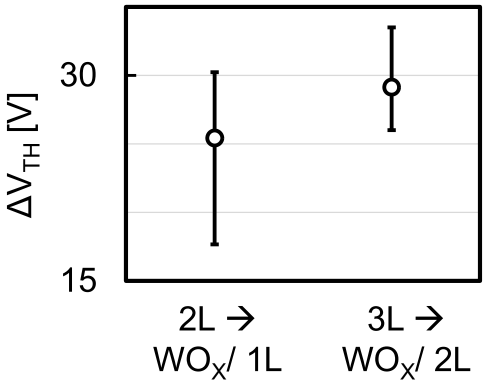

Figure S6. V_{TH} shifting for 2L-/ 3L-WSe_2 FETs after WO_x conversion, indicative of the strength of p-doping effect.

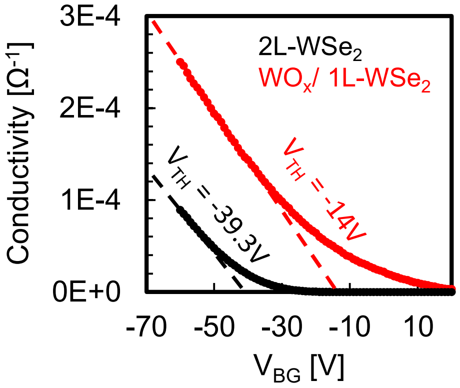

Figure S7. Four-probe extracted channel conductivity versus V_{BG}, enabling the determination of channel V_{TH} excluding the influence from R_C. Apparent V_{TH} shifting results from the p-doping of converted-WO_x.

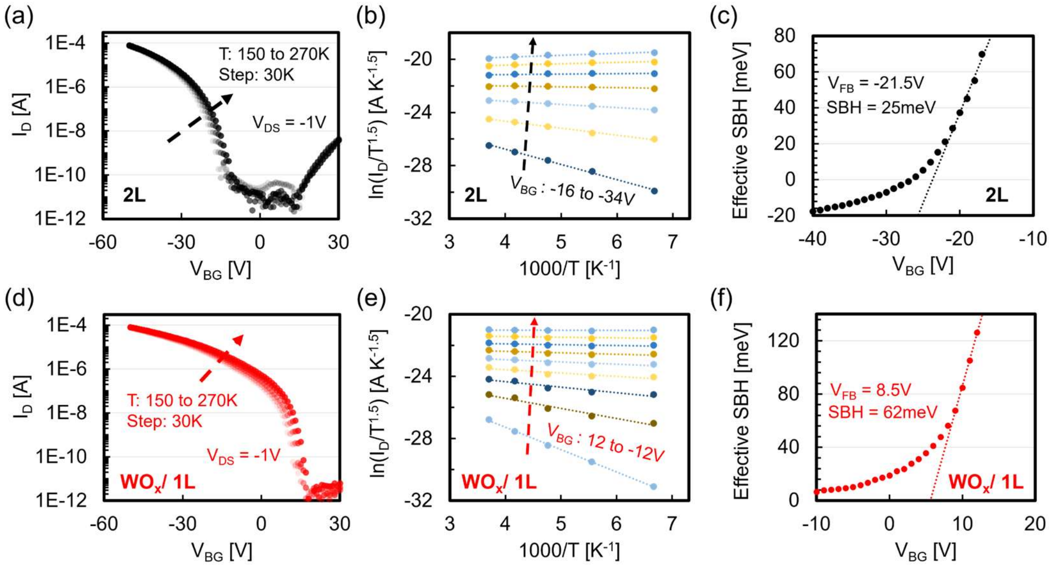

Figure S8. Temperature-dependent I_D-V_{BG} measurement of a 2L-WSe_2 device with Pd contact before/ after converted-WO_x p-doping. (a) Transfer characteristics of the 2L-WSe_2 device at various temperature. (b) Arrhenius plot of thermionic emission in 2L-WSe_2 under various V_{BG}. (c) Effective SBH under various V_{BG} extracted from (b). (d) Transfer characteristics of the WO_x / 1L-WSe_2 device converted from the device in (a) at various temperature. (e) Arrhenius plot of thermionic emission in WO_x / 1L-WSe_2 under various V_{BG}. (f) Effective SBH under various V_{BG} extracted from (e).

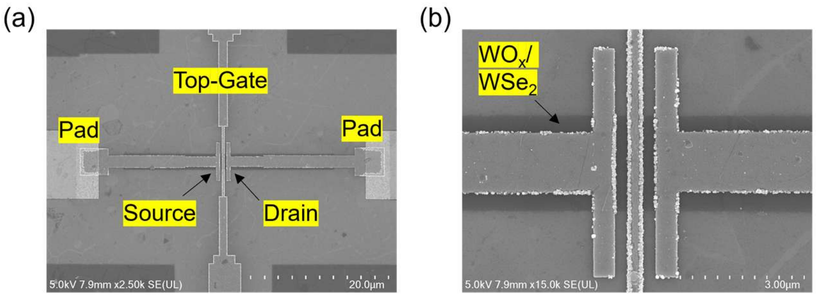

Figure S9. SEM images of the p-MOSFET with 3L-WSe_2 as the channel and WO_x/ 2L-WSe_2 as the spacers. (a) Full view. (b) Magnified image in the active area.
